# Choosing an Appropriate Outlier‐Detection Method When Comparing Institutional Performances in Clinical Audits

**DOI:** 10.1002/bimj.70196

**Published:** 2026-10-22

**Authors:** Anqi Sui, Gareth Ambler, Rumana Z. Omar, Mark de Belder, Menelaos Pavlou

**Affiliations:** ^1^ Department of Statistical Science UCL London UK; ^2^ NHS Arden and Greater East Midlands Commissioning Support Unit Derby UK

**Keywords:** assumption violation, funnel plot, outlier detection, random effects logistic regression

## Abstract

Clinical audits are essential for monitoring and improving the quality of healthcare with statistical outlier detection used to identify healthcare units whose performance deviates from expected standards. Two commonly used outlier‐detection approaches are Common Mean Model (CMM) and Random Effects Logistic Regression (RELR). However, these methods can identify different healthcare units as outliers when applied to the same dataset. Incorrectly flagging a unit as an outlier can have serious consequences, including reputational harm, decreased staff morale, unnecessary regulatory scrutiny, diversion of resources, and loss of public trust. This article investigates the reasons for these discrepancies through a simulation study evaluating the performance of the CMM and RELR under a range of scenarios. The results show that differences in outlier detection are primarily driven by the degree to which a normality assumption holds for each method. When these assumptions are violated, for one or both methods, the two approaches may produce markedly different outlier detection. To choose the most appropriate approach for a given dataset, we recommend assessing each normality assumption, by combining visual diagnostics, such as QQ plots, with quantitative measures, such as Lin's concordance correlation coefficient (CCC).

## 1 Introduction

Clinical audits play a vital role in driving quality improvement within UK healthcare (Care Quality Commission (CQC) 2023; National Institute for Cardiovascular Outcomes Research (NICOR) 2025; Healthcare Quality Improvement Partnership (HQIP) 2024; Paediatric Intensive Care Audit Network (PICANet) 2024; National Clinical Audit Advisory Group 2011). Their primary objective is to evaluate whether the care provided by healthcare units, such as hospitals, general practices, or individual clinicians, adheres to established clinical standards and guidelines. By benchmarking actual practice against these standards, audits help identify outliers, which are healthcare units that demonstrate divergent performance compared to most of the units, either in terms of outcomes (e.g., in‐hospital mortality rates) or processes (e.g., patient waiting times) of clinical care. Identifying potential outliers in audit outcomes can help support the delivery of safe, effective, and high‐quality patient care, inform health policy, guide professional development, and support resource allocation. Misclassifying a healthcare unit as an outlier may lead to significant adverse consequences, such as reputational damage, reduced staff morale, unnecessary regulatory attention, misallocation of resources, and a decline in public trust. Similarly, failing to identify a genuinely underperforming unit can result in ongoing risks to patient safety and quality of care. Given these implications, it is essential that statistical methods for outlier detection in clinical audits are applied appropriately.

Clinical audit data are commonly obtained from multiple healthcare units, with patients hierarchically nested within these higher level units, for example, patients within hospitals, patients operated on by specific surgeons, thereby generating clustered data structures. We focus on scenarios where the patient‐level outcome is binary, for example, alive versus dead. Two important and distinct sources of variability must be considered when comparing unit performance. First, patient outcomes within the same unit are typically more similar to each other than to those from other units, giving rise to between‐unit heterogeneity. When statistical methods are applied to detect outliers in such clustered data, they have to account for this heterogeneity; failure to do so may lead to underestimated variability and incorrect identification of outlying units. Approaches for accounting for clustering will be discussed in Section 2.

Another source of variability arises from differences in patient characteristics across units. Unlike clustering, which reflects correlation within units, this variation reflects differences in patient case‐mix between units. It is therefore important to account for this variation as it can influence unit‐level performance. For example, units that care for sicker or more complex patients may appear to perform worse, primarily due to their patient mix rather than worse quality of care. To account for some of these differences in patient characteristics and to better understand the differences in outcomes that are due to the quality of care provided, risk adjustment should be incorporated (Iezzoni 1994, 2009). Risk adjustment accounts for each patient's predicted risk of experiencing the event of interest (e.g., death). This predicted risk is typically obtained from a risk prediction model (Iezzoni 2009).

Commonly used statistical methods for outlier detection in clinical audits include Common Mean Model (CMM) (PICANet 2024; National Clinical Audit Advisory Group 2011; Grote et al. 2021; Spiegelhalter 2005a; Spiegelhalter et al. 2012; Verburg et al. 2017), also called the “funnel plot” approach, and Random Effects Logistic Regression (RELR) (Austin et al. 2003; Jones and Spiegelhalter 2011; Pavlou et al. 2023). Both methods assume a null distribution for some measure of unit‐level performance. For a given significance level, units whose performance measures align with the null distribution are called “in‐control” units, whereas the rest are flagged as potential outliers, warranting further investigation or recognition as high performers. Although both the CMM and RELR are standard tools, they differ fundamentally in their modeling frameworks. The CMM is based on aggregated unit‐level data and assumes independence across units, with unit‐level performance typically defined on the probability scale. In contrast, RELR is a hierarchical model that operates on patient‐level data, explicitly accounting for clustering, and defines unit‐level performance measures on the logit scale. As shown later (Section 5), applying each method separately to the same data may result in different units being identified as outliers, which could be due to the corresponding distributional assumptions being on different scales. Although the CMM appears to be more widely used in practice (CQC 2023; HQIP 2024; PICANet 2024; National Clinical Audit Advisory Group 2011; New York State Department of Health 2023; Grote et al. 2021), probably due to its ease of implementation and visualization and the availability of software, those inconsistencies in outlier detection highlight the importance of critically evaluating each method for its appropriateness across different scenarios and selecting the one best suited to a specific context.

This article aims to investigate the reasons for those discrepancies and to provide evidence‐based recommendations to guide the selection of appropriate methods for outlier detection in clinical audits. The structure of the article is as follows. In Section 2, we present the CMM and RELR methods in detail. Section 3 clarifies the key distributional assumptions for both methods and describes diagnostic tools to assess how well the corresponding assumptions are satisfied. In Section 4, we use simulation to evaluate and compare the performance of both methods under different scenarios, examining how violation of their assumptions affects their performance. Section 5 illustrates the application of each method to a real‐world dataset. In Section 6, we discuss our findings and provide practical recommendations for method selection.

## 2 Commonly Used Methods

### 2.1 Common Mean Model

Consider a dataset consisting of k units, indexed by i=1,…,k, where unit i contains $n_{i}$ patients, indexed by $j=1,\text{…},\;n_{i}$. Let $Y_{ij}$ denote the binary outcome for patient j in unit i, where $Y_{ij}=1$ is defined as the occurrence of the event of interest and 0 otherwise. The total number of observed events in unit i is given by $O_{i}=\sum_{j=1}^{n_{i}}y_{ij}$, where $y_{ij}$ is the realized value of $Y_{ij}$. Let $p_{i}$ denote the observed performance for unit i, which is often taken to be the proportion of events or risk‐adjusted proportion of events. When a risk model is available, each patient is assigned a predicted risk of having the event, denoted by $p_{ij}^{*}$. The expected number of events in unit i is $E_{i}=\sum_{j=1}^{n_{i}}p_{ij}^{*}$. The risk‐adjusted proportion of events is calculated as the ratio of the observed and expected number of events, multiplied by the average proportion of events across all units: $p_{i}=\left(O_{i}/E_{i}\right)\;\times \left(\sum_{i=1}^{k}O_{i}/\sum_{i=1}^{k}n_{i}\right)$. Without a loss of generality, we use $p_{i}$ to denote the observed performance for unit i, regardless of whether it is risk‐adjusted or not.

The CMM assumes that the observed performance of all units shares a common true performance and that any variation in observed values is due to chance (Spiegelhalter 2005a). Under a normal approximation,

(1) $$p_{i}∼\mathrm{Normal}\left(p,\;p\left(1-p\right)/n_{i}\right),$$

where p represents the underlying true performance, also called the benchmark, and the variance term arises from random binomial variation. The benchmark p can be externally defined, such as using a national standard or an external benchmark derived from previous audits, or it can be estimated from the data. When estimated from the data, it typically is the average performance across all units, for example, the average proportion of events $\;\hat{p}=\sum_{i=1}^{k}O_{i}\;/\sum_{i=1}^{k}n_{i}$. To identify potential outliers, the CMM approach tests the null hypothesis,

$$\;H_{0}:p_{i}=p\mathrm{versus}\;H_{1}:p_{i}\neq p$$

which leads to the construction of the test statistic,

$$\;Z_{i}=\left(p_{i}-p\right)\;/\;\sqrt{p\left(1-p\right)/n_{i}}.$$

However, in practice, the observed variability often exceeds the random binomial variance assumed under the CMM. This extra variability is known as overdispersion (Spiegelhalter 2005a), which usually results from factors, such as clustering and ineffective risk adjustment. To account for overdispersion, the CMM is usually applied together with an overdispersion correction. A commonly used overdispersion correction is the multiplicative overdispersion correction (Spiegelhalter 2005a), which, as the name suggests, multiplies the assumed binomial variance by an overdispersion factor φ often estimated as $\hat{\varphi }=\max\left(1,\;\sum_{i=1}^{k}Z_{i}^{2}/k\right)$ (Spiegelhalter 2005a, 2005b). After correcting for overdispersion, the test statistic for the CMM becomes

(2) $$Z_{i}^{\mathrm{CMM}}=\left(p_{i}-p\right)\;/\;\sqrt{\hat{\varphi }p\left(1-p\right)/n_{i}}.$$

Under the null hypothesis, a unit is identified as an outlier if the absolute value of its test statistic meets or exceeds the critical value $|z_{\alpha /2}|$ for a given significance level α, that is, $|Z_{i}^{\mathrm{CMM}}\left|\geq \right|z_{\alpha /2}|$, where $\alpha /2=P\left(Z_{i}^{\mathrm{CMM}}\leq z_{\alpha /2}\right)=\Phi \left(z_{\alpha /2}\right)$. Failure to account for overdispersion may result in an underestimation of the observed variability, thereby causing an excessively large number of units to be identified as outliers. All subsequent mentions of the CMM in this article refer to the overdispersion‐corrected CMM.

Outlier‐detection results from the CMM can be visualized using a “funnel plot” (Spiegelhalter 2005a; Grote et al. 2021; Verburg et al. 2017), where the observed performance for a given unit $p_{i}$ is plotted against a measure of its precision, such as unit size $n_{i}$. A funnel plot also includes a horizontal line representing the benchmark p, along with “control limits,” which are also known as prediction intervals (Seaton and Manktelow 2012). The control limits for unit i are calculated as (Spiegelhalter 2005a)

$$p\pm z_{\alpha /2}\sqrt{\hat{\varphi }p\;\left(1-p\right)/n_{i}}.$$

That is, for an in‐control unit, the probability that its observed performance lies below the lower control limit is α/2, the probability of exceeding the upper control limit is α/2, and the total probability of being outside the control limits is α. Units with observed performance $p_{i}$ outside the control limits are identified as potential outliers. This is equivalent to identifying outliers by assessing whether the corresponding absolute test statistic exceeds the critical value $|z_{\alpha /2}|$. We present a funnel plot for a real‐world dataset in Section 5.

### 2.2 RELR Model

For hierarchical data, RELR is widely used to model binary outcomes. Through the inclusion of unit‐specific random effects, representing unit‐level variation, RELR explicitly accounts for the clustered nature of the data and thus between‐unit heterogeneity. Unlike the CMM, which operates at the aggregated level, RELR is fitted to patient‐level data and allows risk adjustment to be directly incorporated through covariates. A simple form of the RELR model is

$$\begin{array}{ccc} \mathrm{logit}\;\left(P\left(\;Y_{ij}=1\right)\right) & = & \log\;\left(P\left(\;Y_{ij}=1\right)/\left(1-P\left(\;Y_{ij}=1\right)\right)\right)\\ & = & \beta_{0}+\beta_{1}\mathrm{logit}\left(p_{ij}^{*}\right)+u_{i}, \end{array}$$

where $\beta_{0}$ is the fixed intercept, and $\beta_{1}$ is the regression coefficient associated with the logit of an individual patient's predicted risk $p_{ij}^{*}$. The latter coefficient is usually included to account for potential miscalibration (or “drift”) of the risk model (Harrell et al. 1996). If no risk adjustment is used, the term $\mathrm{logit}(p_{ij}^{*})$ is omitted from the model ($\beta_{1}=0$). The random effects $u_{i}$’s represent the unit‐specific deviation from the fixed intercept $\beta_{0}$. Usually, it is assumed that $u_{i}∼\mathrm{Normal}\left(0,\;\sigma_{u}^{2}\right)$, where $\sigma_{u}^{2}$ is the between‐unit variance (Goldstein 2011). The proportion of total variation that is attributable to the variance between units, as opposed to within‐units, can be quantified by the intraclass correlation coefficient (ICC) (Eldridge et al. 2009). The ICC can be approximated as (Eldridge et al. 2009)

(3) $$\mathrm{ICC}\;=\sigma_{u}^{2}\;/\;\left(\sigma_{u}^{2}+\pi^{2}/3\right),$$

which ranges from 0 to 1. Higher ICC values indicate greater between‐unit variance relative to within‐unit variance. In studies with data clustered by units, in contrast to longitudinal data, the ICC is typically low, often below 0.10 (Adams et al. 2004; Gulliford et al. 1999). The RELR model presented above can be fitted in standard software (e.g., the function glmer in package lme4 in R) to obtain estimates of the fixed and random effects.

To identify outliers, the RELR tests the null hypothesis that the random effects of all units come from the assumed null distribution, $H_{0}:u_{i}∼\mathrm{Normal}\left(0,\;\sigma_{u}^{2}\right)$. To test the null hypothesis, the following test statistics are constructed:

(4) $$Z_{i}^{\mathrm{RELR}}={\hat{u}}_{i}/SE^{D}\left({\hat{u}}_{i}\right)\;,$$

where ${\hat{u}}_{i}$’s are estimates of the random effects, typically obtained using Empirical Bayes (EB) prediction, and $SE^{D}\left({\hat{u}}_{i}\right)$ denotes the diagnostic standard error of the estimated random effect ${\hat{u}}_{i}$. We note that the diagnostic standard error is the appropriate type of standard error used to characterize the uncertainty of the EB estimated random effects for the purpose of outlier detection; these can be obtained via an approximation method or nonparametric bootstrapping, as described by Skrondal and Rabe‐Hesketh (2009). A unit is flagged as an outlier by the RELR method if its test statistic is as extreme as or more extreme than a critical value, $|Z_{i}^{\mathrm{RELR}}\left|\geq \right|z_{\alpha /2}|$, suggesting that the random effect for unit i is unlikely to come from the assumed null distribution for the random effects.

## 3 Assessing the Normality Assumptions for the CMM and RELR Approaches

As shown in Section 2, both the CMM and RELR methods require normality assumptions, but their assumptions pertain to different sets of test statistics. The CMM assumes normality for $Z_{i}^{\mathrm{CMM}}$ in Equation (2), which are constructed on the basis of differences between the observed and expected event proportions and are therefore on the *probability* scale. In contrast, the RELR assumes normality for $Z_{i}^{\mathrm{RELR}}$ in Equation (4), which are derived from the estimates of the random effects and therefore lie on the *logit* scale. The relationship between a probability and its logit value is approximately linear when the probability is around 0.5 but becomes extremely nonlinear when the probability approaches either 0 or 1 (Appendix A.1). Consequently, the normality assumptions of the two methods cannot both be satisfied across the entire range of probability. In settings with low event prevalence and/or large between‐unit heterogeneity, some unit‐level proportions will be far from 0.5, and we might expect different outlier‐detection results between the two methods.

To choose the most suitable method for a given dataset, it is essential to assess the normality assumption for each set of the test statistics and ultimately select the method whose normality assumption is better supported. A common approach to assessing normality is a normal Quantile–Quantile (QQ) plot, where the quantiles of the observed test statistics are plotted against the corresponding quantiles of the standard normal distribution. If the test statistics are consistent with the standard normal distribution, the points in the QQ plot should approximately fall on a reference line with slope 1 and intercept 0 (line of equality). However, QQ plots are usually visually inspected, and their interpretation is often subjective. To measure the agreement with normality quantitatively, we additionally use Lin's concordance correlation coefficient (CCC), which evaluates the agreement between the (paired) measurements from two variables by measuring their deviation from the line of equality (Lin, 1989). In our context, Lin's CCC can take values between 0 and 1; the higher the Lin's CCC, the better the agreement with normality.

## 4 Simulations

We outline the design of our simulation study based on the ADEMP reporting framework (Morris et al. 2019) and subsequently present the simulation results. All analyses were conducted in R (version 4.4.1; R Core Team 2024). RELR models were fitted using the lme4 package (Bates et al. 2015). Visualizations were created using ggplot2 (Wickham 2011).

### 4.1 Data‐Generating Mechanisms (DGMs)

We consider two DGMs, corresponding to the CMM and the RELR model, respectively, each examined under scenarios with and without outliers. Without a loss of generality, we attribute all variability in outcomes to differences between units and therefore do not incorporate risk adjustment in our simulation.

In the CMM DGM, we first simulate the observed event proportions for in‐control units following the CMM given in Equation (1), with a prespecified overdispersion factor φ≥1 and a prespecified outcome prevalence p. For outliers, the observed event proportions are generated from

$$\mathrm{Normal}\left(p\pm 2.3\sqrt{\varphi p\left(1-p\right)/n_{i}},\;10^{-6}\right),$$

with their means shifted by ±2.3 standard deviations relative to the in‐control distribution, which corresponds to a two‐sided significance level of 2%, reflecting moderately extreme but plausible deviations that may occur in practice. The small variance of $10^{-6}$ is used to minimize overlap with the in‐control units. To ensure that all generated values represent valid probabilities, any proportions below 0 are set to 0, and any proportions above 1 are set to 1. To obtain patient‐level binary outcomes, an outcome value of 1 is assigned to $O_{i}$ patients and 0 to the remaining $\left(n_{i}-O_{i}\right)$ patients.

In the RELR–DGM, we first simulate the random effects for in‐control units from $\mathrm{Normal}(0,\;\sigma_{u}^{2})$, where $\sigma_{u}^{2}$ is calculated from a prespecified ICC using Equation (3). Random effects for outliers are simulated from a normal distribution centered ±2.3 standard deviations relative to the mean of the in‐control distribution: $\mathrm{Normal}(\pm 2.3\sigma_{u},\;10^{-6})$, again with a small variance to limit overlap. Patient‐level binary outcomes in unit i are drawn from a Bernoulli distribution with probability given by $\mathrm{logi}t^{-1}\left(\beta_{0}+u_{i}\right)$. The value of $\beta_{0}$ is obtained by optimization so that the numerically evaluated integral $\int_{-\infty }^{\infty }\mathrm{logi}t^{-1}\left(\beta_{0}+u_{i}\right)\phi \left(u\right)du$ equals a target prevalence, where ϕ(u) denotes the density of the normal random variable with mean 0 and variance $\sigma_{u}^{2}$.

In our simulation, we vary the number of units, unit size, proportion of outliers, outcome prevalence, the overdispersion factor for the CMM–DGM, and the ICC values for the RELR–DGM. Table 1 summarizes the parameters varied in our simulation, with each combination constituting a separate scenario. Note that values of the overdispersion factor and ICC are not all comparable as they relate to different models.

**TABLE 1:** Parameters varied in the simulation.

Parameters	Values
Number of units	50, 100, 200
Unit size	500, 1000, 2000
Outlier proportion	0%, 4%, 8%
Outcome prevalence	0.02, 0.05, 0.10, 0.20, 0.30, 0.40, 0.50
Overdispersion factor for the CMM–DGM	2, 4, 8
Intraclass correlation coefficient for the RELR–DGM	0.03, 0.05, 0.10

Abbreviations: CMM, common mean model; DGM, data‐generating mechanism; RELR, random effects logistic regression.

### 4.2 Methods

The outlier‐detection process is applied to each simulated dataset in the following three ways: (i) the CMM method; (ii) the RELR method; and (iii) our proposed approach, referred to as QQ–Lin, which chooses the method that yields the highest value of Lin's CCC.

### 4.3 Simulation Performance Measures

The performance of the CMM and RELR methods is assessed using the false positive rate (FPR) and sensitivity. The FPR is defined as the proportion of in‐control units that are incorrectly detected as outliers, either with significantly lower (“good” outliers) or higher (“bad” outliers) unit‐level performance, relative to the total number of in‐control units. In our simulation, the significance level is set to α=0.05 for detecting outliers on both sides of the test statistics distribution. Consequently, the nominal FPR for each side (good or bad outliers) is 0.025, whereas the nominal overall FPR level is 0.05. We examine the FPR both overall and separately for good and bad outliers.

Sensitivity is defined as the proportion of outliers correctly identified among all true outliers. Together with FPR, the two measures are chosen to reflect key practical considerations in clinical audits. Given the significant consequences of misclassifying in‐control units as outliers in clinical settings, control of FPR is of primary importance to keep the proportion of such misclassifications within acceptable levels. A method is not considered valid unless its overall and separate FPRs (for good and bad outliers) under the null are controlled at the nominal levels. Among methods with well‐controlled FPRs, preference is then given to those with higher sensitivity, reflecting a stronger ability to detect true outliers.

Additionally, we evaluate the agreement rate between the CMM and RELR methods in outlier detection. Every unit in each simulated dataset is assigned an indicator that equals 1 if the two methods produce matching classifications and 0 otherwise. For each simulated dataset, we compute an overall agreement rate, which is the proportion of units with matching classifications between the two methods, and an outlier agreement rate, which is the proportion of outliers with matching classifications. Scenario‐specific agreement rates are then obtained by averaging these agreement rates across all simulated datasets.

For QQ–Lin, we also calculate the proportion of times it correctly chooses the method that aligns with a given DGM. We simulate 500 datasets for each scenario, which results in a small Monte Carlo standard error (MCSE) for the estimates of all performance measures (Table 2).

**TABLE 2:** Largest Monte Carlo standard errors (MCSE) for each simulation performance measure across 500 simulations.

Performance measure	MCSE
False positive rate	≤0.003
False positive rate for good outliers	≤0.003
False positive rate for bad outliers	≤0.001
Sensitivity	≤0.017
Overall agreement rate	≤0.003
Agreement rate for outliers	≤0.016
Proportion of correct selections by QQ–Lin	≤0.022

Abbreviation: QQ, Quantile‐Quantile.

### 4.4 Results

Figures 1 and 2 present the overall FPR of all three methods when data are generated from the CMM– and RELR–DGM, respectively. When there are no outliers, all three methods provide adequate control of the overall FPR in both DGMs, which remains at or close to the nominal level of 0.05. However, a closer examination of the separate FPRs for good and bad outliers under both DGMs (Figures 3 and 4) reveals deviations from the nominal level when the outlier‐detection method does not align with the underlying DGM. Specifically, when the outcome prevalence is close to 0, applying the RELR detection method to data generated under the CMM–DGM leads to over‐detection of good outliers and under‐detection of bad outliers (Figure 3); and vice versa (Figure 4). When the outcome prevalence is close to 1, the reverse pattern occurs due to the shape of the logit function. These patterns become more pronounced with greater variability between units (φ or ICC). This is because with higher variability, a larger number of units have event proportions close to 0 or 1, that is, the regions where the logit function is highly nonlinear. It is important to note that the overall and separate FPRs of QQ–Lin closely match those of the method that is correctly aligned with a given DGM in both CMM– and RELR–DGM settings.

**FIGURE 1:**
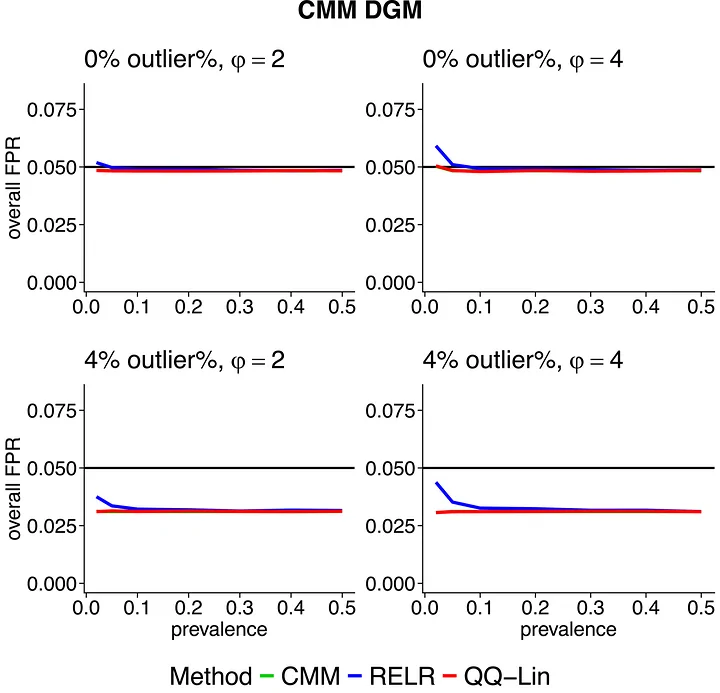
Overall false positive rate (FPR) under CMM–DGM when there are 100 units of size 1000, across scenarios varying in outlier percentage and overdispersion factor φ. The horizontal black line represents the nominal level of 0.05 for the overall FPR. CMM, common mean model; DGM, data‐generating mechanism; QQ, Quantile–Quantile; RELR, random effects logistic regression.

**FIGURE 2:**
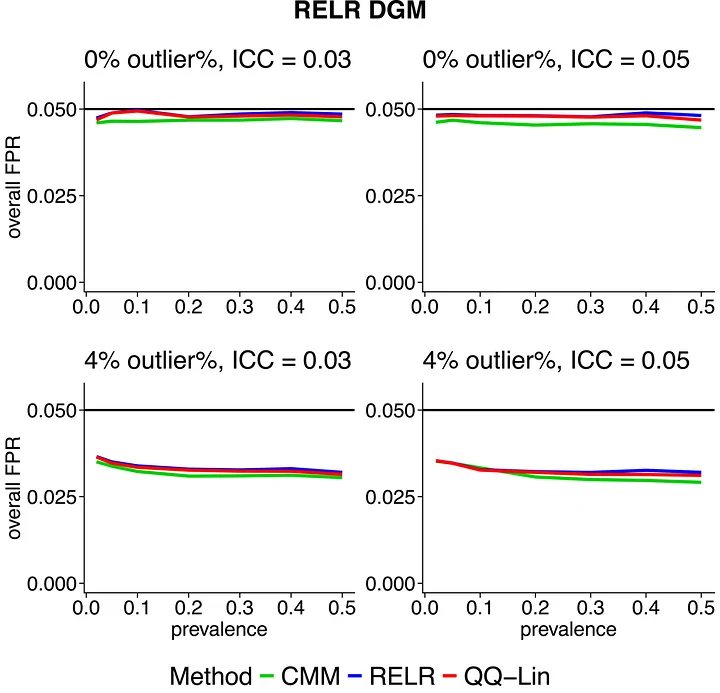
Overall false positive rate (FPR) under RELR–DGM when there are 100 units of size 1000, across scenarios varying in outlier percentage and intraclass correlation coefficient (ICC). The horizontal black line represents the nominal level of 0.05 for the overall FPR. CMM, common mean model; DGM, data‐generating mechanism; QQ, Quantile–Quantile; RELR, random effects logistic regression.

**FIGURE 3:**
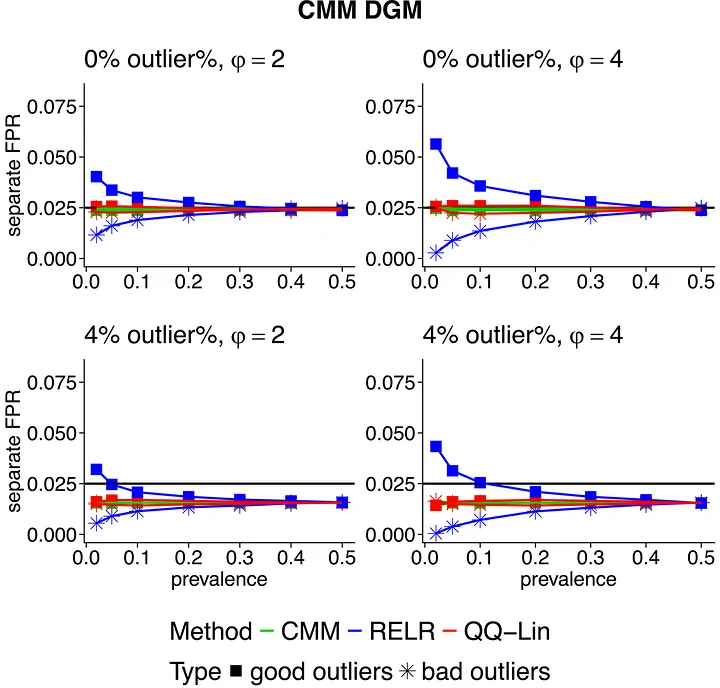
Separate false positive rate (FPR) for good and bad outliers under CMM–DGM when there are 100 units of size 1000, across scenarios varying in outlier percentage and overdispersion factor φ. The horizontal black lines represent the nominal level of 0.025 for the separate FPRs. CMM, common mean model; DGM, data‐generating mechanism; QQ, Quantile–Quantile; RELR, random effects logistic regression.

**FIGURE 4:**
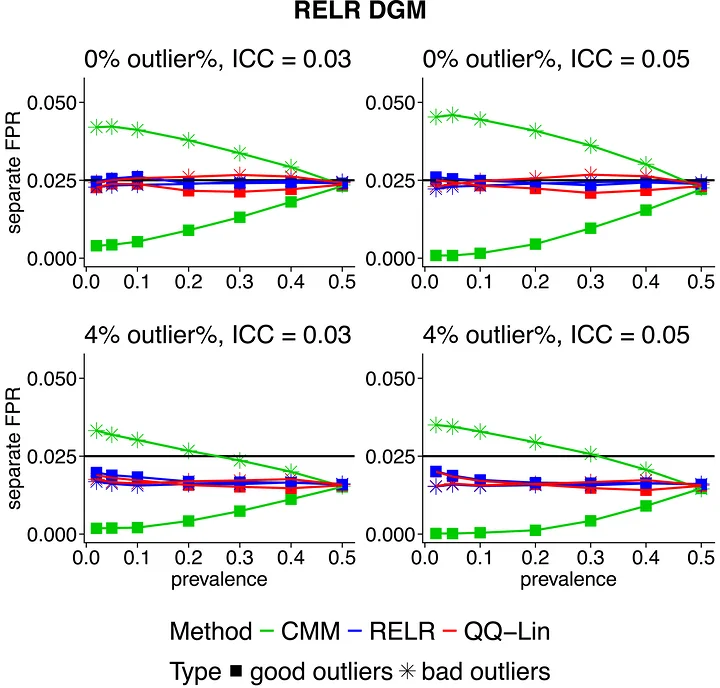
Separate false positive rate (FPR) for good and bad outliers under RELR–DGM when there are 100 units of size 1000, across scenarios varying in outlier percentage and intraclass correlation coefficient (ICC). The horizontal black lines represent the nominal level of 0.025 for the separate FPRs. CMM, common mean model; DGM, data‐generating mechanism; QQ, Quantile–Quantile; RELR, random effects logistic regression.

Figures 5 and 6 show the sensitivity to detect outliers of the three methods under CMM– and RELR–DGM settings, respectively. QQ–Lin shows sensitivity that closely matches the method that aligns with a given DGM. As the outlier proportion increases, the overall and separate FPRs of all three methods fall further below the nominal levels, and their sensitivity decreases. This is because the outliers, which have more extreme observed values, inflate the estimates of the overdispersion factor in the CMM method or the between‐unit variance in the RELR method. This reduces the chance of identifying outliers, reflected by the FPRs lower than nominal and reduced sensitivity. Several robust estimation techniques have been proposed to account for the effect of outliers, including Winsorization (Spiegelhalter 2005a) and cross‐validation (Ohlssen et al. 2007). As this is not the focus of this study, we do not discuss them further. Additional plots covering all simulation scenarios are provided in Figures S1–S6.

**FIGURE 5:**
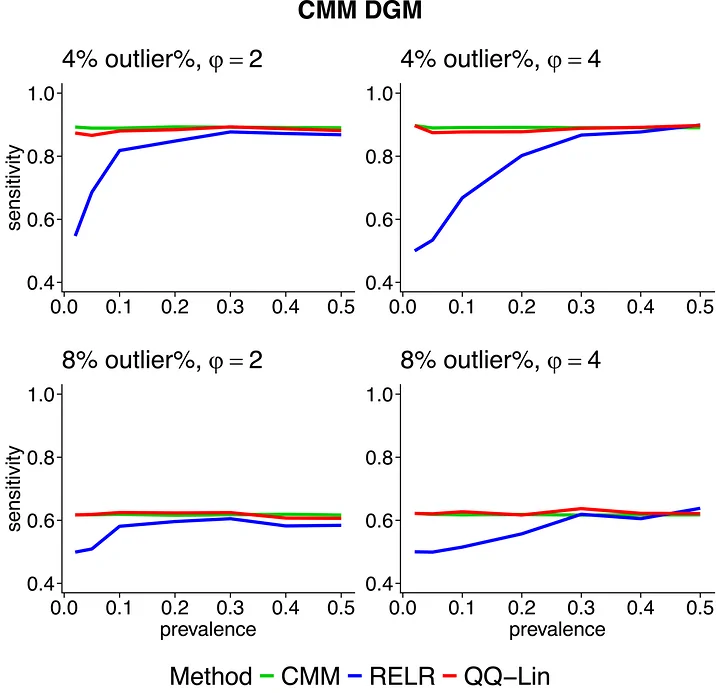
Sensitivity for detection of outliers under CMM–DGM when there are 100 units of size 1000, across scenarios varying in outlier percentage and overdispersion factor φ. CMM, common mean model; DGM, data‐generating mechanism; QQ, Quantile–Quantile; RELR, random effects logistic regression.

**FIGURE 6:**
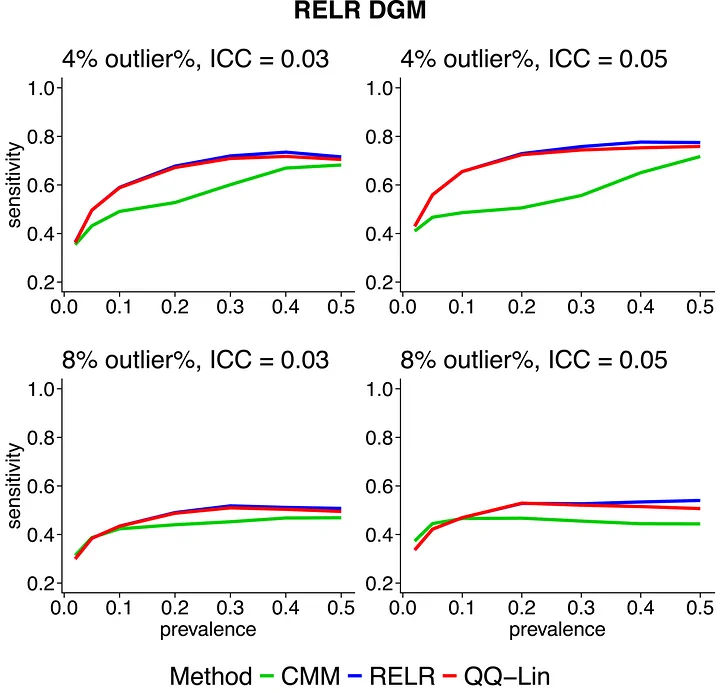
Sensitivity for detection of outliers under RELR–DGM when there are 100 units of size 1000, across scenarios varying in outlier percentage and intraclass correlation coefficient (ICC). CMM, common mean model; DGM, data‐generating mechanism; QQ, Quantile–Quantile; RELR, random effects logistic regression.

Table 3 presents the proportion of times that QQ–Lin selects the correct outlier‐detection method for a given DGM when there are 100 units of size 1000. Results for different combinations of numbers of units and unit sizes are provided in Tables S1 and S2. Overall, the proportion of correct selections by QQ–Lin is little affected by the numbers of units and unit sizes investigated. Even under low‐prevalence scenarios (≤0.10), where the choice of method is more critical, QQ–Lin achieves a proportion of correct selections of at least 0.73. As the outcome prevalence approaches 0.5, the proportion of correctly selected methods generally declines. This pattern is expected, as when prevalence is near 0.5, the distinction between the CMM and RELR methods becomes less consequential than at prevalences closer to 0 or 1, where method choice has a greater impact on outlier detection. This is also reflected in the outlier (Table 4) and overall (Table S3) agreement rates between the two methods. As prevalence approaches 0.5, the outlier‐detection results from the two methods become increasingly similar, resulting in higher agreement rates.

**TABLE 3:** Proportion of correctly selected methods by QQ–Lin (500 simulations; 100 units of size 1000).

CMM–DGM								
Overdispersion factor	Outlier%	Prevalence						
		0.02	0.05	0.10	0.20	0.30	0.40	0.50
2	0	0.86	0.76	0.68	0.62	0.61	0.63	0.78
	4	0.95	0.84	0.74	0.65	0.61	0.62	0.75
	8	0.98	0.89	0.80	0.69	0.63	0.60	0.63
4	0	0.97	0.86	0.78	0.66	0.59	0.55	0.73
	4	1.00	0.95	0.88	0.72	0.65	0.59	0.69
	8	1.00	0.99	0.92	0.79	0.68	0.61	0.71
8	0	1.00	0.97	0.87	0.75	0.64	0.57	0.66
	4	1.00	1.00	0.96	0.86	0.71	0.62	0.70
	8	1.00	1.00	0.99	0.89	0.77	0.64	0.67

RELR–DGM								
Intraclass correlation coefficient	Outlier%	Prevalence						
		0.02	0.05	0.10	0.20	0.30	0.40	0.50
0.03	0	0.93	0.93	0.90	0.86	0.76	0.64	0.46
	4	0.96	0.97	0.95	0.92	0.84	0.69	0.47
	8	0.98	0.98	0.98	0.96	0.88	0.71	0.55
0.05	0	0.98	0.98	0.94	0.91	0.82	0.69	0.50
	4	1.00	0.99	0.99	0.97	0.90	0.75	0.52
	8	1.00	1.00	1.00	0.99	0.94	0.77	0.60
0.10	0	1.00	1.00	0.99	0.97	0.91	0.79	0.62
	4	1.00	1.00	1.00	1.00	0.96	0.84	0.64
	8	1.00	1.00	1.00	1.00	0.99	0.87	0.71

Abbreviations: CMM, common mean model; DGM, data‐generating mechanism; RELR, random effects logistic regression.

**TABLE 4:** Mean (standard deviation) agreement rates in outlier classification between the common mean model (CMM) and random effects logistic regression (RELR) approaches among outlying units (500 simulations; 100 units of size 1000).

CMM–DGM								
Overdispersion factor	Outlier%	Prevalence						
		0.02	0.05	0.10	0.20	0.30	0.40	0.50
2	4	0.55 (0.23)	0.69 (0.30)	0.83 (0.28)	0.90 (0.23)	0.94 (0.17)	0.97 (0.12)	0.98 (0.11)
	8	0.50 (0.31)	0.56 (0.33)	0.65 (0.33)	0.78 (0.29)	0.87 (0.22)	0.93 (0.17)	0.97 (0.13)
4	4	0.48 (0.19)	0.53 (0.23)	0.67 (0.29)	0.82 (0.28)	0.91 (0.22)	0.96 (0.15)	0.99 (0.06)
	8	0.46 (0.31)	0.50 (0.31)	0.53 (0.32)	0.65 (0.33)	0.78 (0.29)	0.89 (0.21)	0.98 (0.10)
8	4	0.02 (0.10)	0.50 (0.18)	0.52 (0.21)	0.69 (0.29)	0.83 (0.27)	0.94 (0.18)	0.99 (0.06)
	8	0.06 (0.17)	0.51 (0.31)	0.50 (0.31)	0.55 (0.33)	0.68 (0.33)	0.84 (0.24)	0.97 (0.11)

RELR–DGM								
Intraclass correlation coefficient	Outlier%	Prevalence						
		0.02	0.05	0.10	0.20	0.30	0.40	0.50
0.03	4	0.79 (0.22)	0.69 (0.22)	0.64 (0.24)	0.65 (0.24)	0.72 (0.23)	0.85 (0.18)	0.97 (0.09)
	8	0.75 (0.16)	0.66 (0.18)	0.62 (0.19)	0.62 (0.19)	0.69 (0.17)	0.82 (0.14)	0.96 (0.07)
0.05	4	0.71 (0.23)	0.62 (0.25)	0.60 (0.23)	0.58 (0.23)	0.62 (0.23)	0.78 (0.22)	0.94 (0.12)
	8	0.68 (0.18)	0.59 (0.19)	0.56 (0.20)	0.56 (0.21)	0.59 (0.20)	0.74 (0.16)	0.90 (0.11)
0.10	4	0.67 (0.24)	0.63 (0.24)	0.60 (0.22)	0.55 (0.22)	0.54 (0.21)	0.64 (0.22)	0.83 (0.20)
	8	0.60 (0.20)	0.55 (0.22)	0.54 (0.22)	0.53 (0.23)	0.53 (0.23)	0.65 (0.20)	0.77 (0.16)

Abbreviation: DGM, data‐generating mechanism.

## 5 Illustration: 30‐Day Mortality Following Percutaneous Coronary Intervention (PCI)

The real dataset used in this section includes patients who underwent PCI procedures at 88 hospitals in the United Kingdom over a 3‐year period, from April 2015 to March 2018. Hospital names are replaced with numbers from 1 to 88 for confidentiality. The total number of procedures across all hospitals is 262,035. The median number of procedures per hospital (interquartile range) is 2536 (1383–4270). The outcome of interest is the 30‐day risk‐adjusted mortality following PCI. The British Cardiovascular Intervention Society (BCIS) logistic regression model (Mcallister et al. 2016) is used to obtain the predicted risk of 30‐day mortality for risk adjustment. The median number of observed and expected deaths per hospital (interquartile range) are 70 (21–133) and 58 (18–108), respectively. The average risk‐adjusted mortality rate across all hospitals is 0.034. The estimated overdispersion factor and ICC are 4.45 and 0.033, respectively.

Figure 7 presents a funnel plot for the CMM for the 30‐day risk‐adjusted mortality. The CMM identifies four outliers: Hospitals 10 and 13 with significantly lower mortality, and Hospitals 44 and 12 with significantly higher mortality. Table 5 compares the outlier‐detection results from the CMM and RELR methods. Compared to the CMM, RELR identifies five additional good outliers (Hospitals 33, 36, 46, 58, and 86) while detecting only one bad outlier (Hospital 44).

**FIGURE 7:**
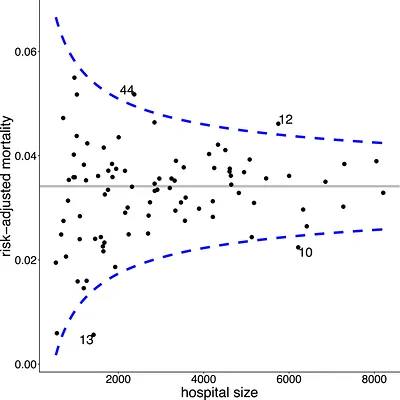
Funnel plot for 30‐day risk‐adjusted mortality in the PCI data (88 hospitals). The grey horizontal line represents the average risk‐adjusted mortality of 0.034 across all hospitals. The blue dashed lines represent the control limits after multiplicative overdispersion correction (at a significance level of 0.05).

**TABLE 5:** Hospitals in the percutaneous coronary intervention (PCI) data with significantly outlying risk‐adjusted mortality identified by the common mean model (CMM) and random effects logistic regression (RELR) methods.

Method	Hospital ID	Risk‐adjusted mortality	Test statistic z	Lower tail probability, Φ(z) ^a^	Outlier type
CMM	13	0.006	−2.806	0.003	Good
	10	0.022	−2.416	0.008	Good
	12	0.046	2.375	0.991	Bad
	44	0.052	2.243	0.988	Bad
RELR	13	0.006	−3.334	<0.001	Good
	46	0.006	−2.738	0.003	Good
	86	0.019	−2.381	0.009	Good
	33	0.016	−2.209	0.014	Good
	36	0.016	−2.201	0.014	Good
	58	0.015	−2.169	0.015	Good
	44	0.052	2.096	0.982	Bad

^a^ For a two‐sided significance level of 0.05, a unit is identified as an outlier if its lower tail probability of the standard normal distribution Φ(z) ≤0.025 (“good” outlier) or ≥0.975 (“bad” outlier).

We assess the normality of the test statistics from the CMM and RELR both visually and quantitatively. Figure 8 presents QQ plots for each set of the test statistics. Table 6 shows quantitative measures of normality. Both Figure 8 and Table 6 suggest that the distribution of the test statistics from the CMM adheres more closely to normality than that from RELR. This indicates that the CMM is more likely to be a more appropriate outlier‐detection method than RELR for the PCI dataset, and that the use of RELR would lead to over‐detection of good outliers and under‐detection of bad outliers. Overall, these results are consistent with our simulation findings.

**FIGURE 8:**
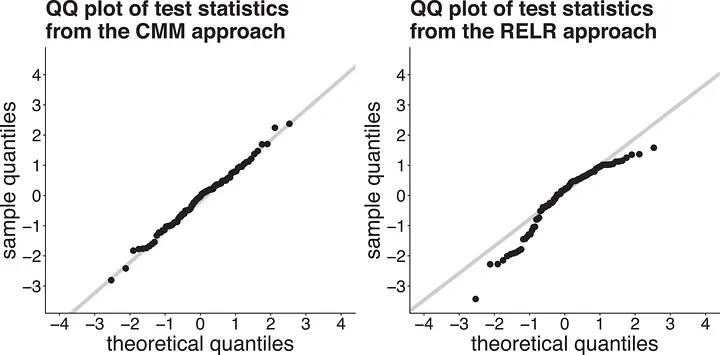
QQ plots for the two sets of test statistics, obtained from the CMM (left) and RELR (right) methods respectively, for the PCI data. CMM, common mean model; QQ, Quantile–Quantile; RELR, random effects logistic regression.

**TABLE 6:** Skewness, kurtosis, and Lin's concordance correlation coefficient (CCC) for the test statistics obtained from the common mean model (CMM) and random effects logistic regression (RELR) approaches for the percutaneous coronary intervention (PCI) data.

	Skewness	Kurtosis	Lin's CCC
Test statistics from CMM	−0.07	3.03	0.99
Test statistics from RELR	−0.70	3.28	0.97

*Note:* If the test statistics follow a normal distribution, the expected values are 0 for skewness and 3 for kurtosis.

## 6 Discussion

Outlier detection is an important process in clinical audits, where accurate identification of outlying healthcare units is essential for maintaining and improving the quality of patient care. Misclassification, either by incorrectly flagging in‐control units as outliers or by failing to detect true outliers, can have serious implications in clinical settings, including unwarranted scrutiny, reputational harm, and missed opportunities for quality improvement.

Two commonly used methods for outlier detection are the CMM and RELR approaches. The CMM, which operates on unit‐level data, is widely adopted due to its ease of implementation, straightforward visualization, and the availability of supporting software. RELR operates on patient‐level data, which may be computationally expensive for very large datasets. In addition, convergence problems may arise when the outcome is rare or when many units have very small unit sizes (Heinze and Schemper 2002). However, it can incorporate risk adjustment via covariates, allowing potential miscalibration of an external risk model to be addressed. Both methods can account for between‐unit heterogeneity: RELR does so through the inclusion of random effects, whereas the CMM can be adapted to accommodate extra variability through an overdispersion correction. Both methods make normality assumptions, but on different scales: the CMM on the probability scale and RELR on the logit scale. Due to the nonlinearity of the logit function, these assumptions cannot hold simultaneously, particularly when the outcome prevalence is near 0 or 1, or when the variability between units is large.

In this study, we reviewed both methods and conducted a simulation study to evaluate their performance across a range of scenarios. To our knowledge, the impact of violations of method assumptions on the performance of the CMM and RELR has not been explored in the literature. Our simulation results indicate that, when prevalence is below 0.5, applying the RELR method to data generated following the CMM can lead to an over‐detection of good outliers and an under‐detection of bad outliers, and the reverse occurs when applying the CMM method to data generated following RELR. These discrepancies become more pronounced as the prevalence approaches 0 or 1, or when the variability between units increases. These patterns are consistent with the discrepancies observed in our real data application.

We showed that visual tools, such as QQ plots, combined with quantitative diagnostics, such as Lin's CCC, can effectively assess the normality assumptions and guide the choice between the CMM and RELR. The performance of QQ–Lin was little affected by the numbers of units and unit sizes investigated. It is straightforward to implement and can be easily incorporated into standard outlier‐detection workflows. Although estimates of Lin's CCC are expected to be less precise when the number of units is small, this uncertainty can be quantified through confidence intervals (Lin 1989). When the outcome prevalence is approximately 0.5, and the variability between units is small, the distinction between the CMM and RELR approaches is less consequential, as expected. Nevertheless, the use of these normality diagnostics is still recommended to support principled and consistent method selection, even though the impact of choosing a suboptimal approach is relatively small in this scenario. In practice, patient‐level data may not always be available, in which case the CMM is the only feasible approach. Even then, it remains important to evaluate the plausibility of the normality assumption using QQ plots and Lin's CCC. When normality appears questionable, results should be interpreted with caution. R codes used for the CMM, RELR, and QQ–Lin are available at the following GitHub repository: https://github.com/asxx08/manuscript‐outlier‐detection.

This study has several limitations. First, we focused only on the two most commonly used methods. Although this choice reflects current practice and allows for a detailed comparison, there may be other methods or variations of the two methods that we have not investigated. Future research could investigate methods that can perform well under both DGMs, potentially reducing the need for method selection. Second, the variability between units is typically estimated directly from the observed data; however, the presence of extreme values or potential outliers can substantially inflate these estimates and thus influence outlier‐detection results. Although robust estimation procedures, such as Winsorization (Spiegelhalter 2005a) or cross‐validation approaches (Ohlssen et al. 2007), could help mitigate the influence of outliers, evaluating these techniques was beyond the scope of this article. Future studies could explore how incorporating robust methods affects the performance of the three methods in terms of outlier detection. Third, our simulation specified that all variability in the outcome was due to differences between units and consequently did not require risk adjustment. In practice, the adequacy of risk adjustment can affect the estimation of unit‐level performance, and consequently, the detection of outliers. Understanding how different levels of risk‐adjustment quality influence the performance of the three methods might provide valuable insights for applied clinical audit settings.

In settings where many (e.g., several hundreds) units are being compared, a large number of outliers may arise purely by chance, due to multiple testing. Though we do not incorporate such adjustments in the present study, this issue can be addressed separately as a post‐processing step (Spiegelhalter 2005a; Pavlou et al. 2023).

To conclude, our findings indicated that the choice between using the CMM and RELR methods should be made carefully as this can directly affect which units are detected as outliers, particularly in clinical settings with low‐prevalence outcomes. Attention should be given to the normality assumptions that underpin both methods and to assessing whether these assumptions are appropriate for the data at hand. Such consideration should lead to more accurate outlier detection and ultimately better inform decision‐making in clinical audits.

## Conflicts of Interest

The authors declare no conflicts of interest.

## Supporting information




**Supporting File:** bimj70196‐sup‐0001‐SuppMat.pdf.

## Figures and Tables

**FIGURE A1:**
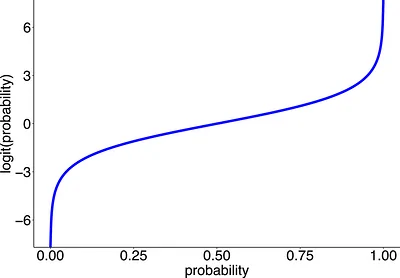
Relationship between probability and its logit transformation.

## Data Availability

The data that support the findings of this study are available from National Institute for Cardiovascular Outcomes Research (NICOR). Restrictions apply to the availability of these data, which were used under license for this study. Data are available from Menelaos Pavlou (m.pavlou@ucl.ac.uk) with the permission of NICOR.

## 1

A.1. Shape of the logit function (Figure A1).
