# Supplementary material for: Choosing an Appropriate Outlier‐Detection Method When Comparing Institutional Performances in Clinical Audits
Source: Biom J. 2026 Oct 22;68(6):e70196. doi: 10.1002/bimj.70196 (PMC13688080; doi:10.1002/bimj.70196)
Supplement: Supplementary file 1 [file BIMJ-68-e70196-s001.pdf]

Supplementary Materials

Sensitivity, overall and separate false positive rates in more simulation scenarios (Figures S1-S6)

Figure S1 Overall false positive rate (FPR) under CMM DGM when there are 100 units of size 1000, across scenarios varying in outlier percentage and overdispersion factor φ. The horizontal black line represents the nominal level of 0.05 for the overall FPR.

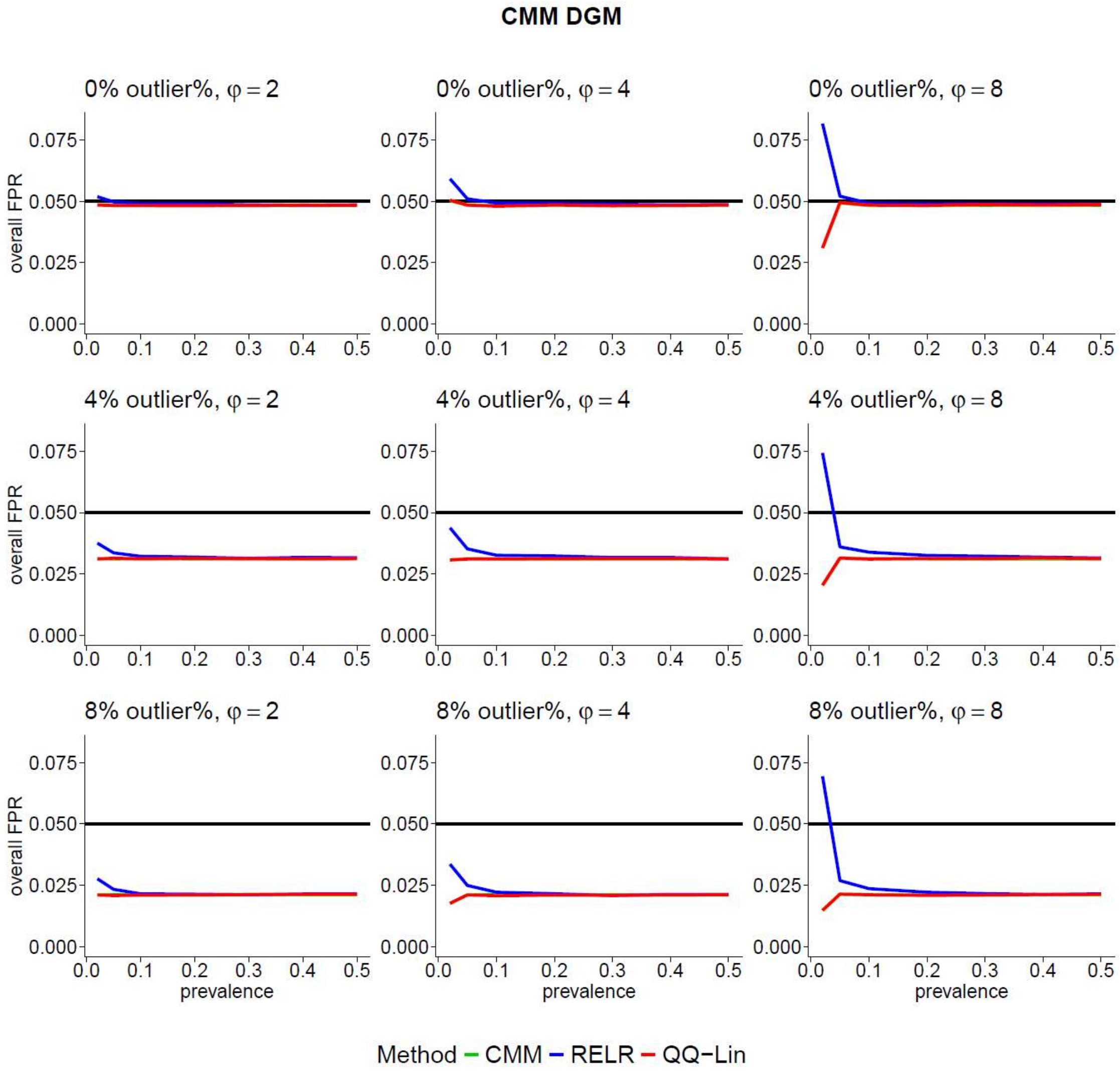

Figure S2 Overall false positive rate (FPR) under RELR DGM when there are 100 units of size 1000, across scenarios varying in outlier percentage and intraclass correlation coefficient (ICC). The horizontal black line represents the nominal level of 0.05 for the overall FPR.

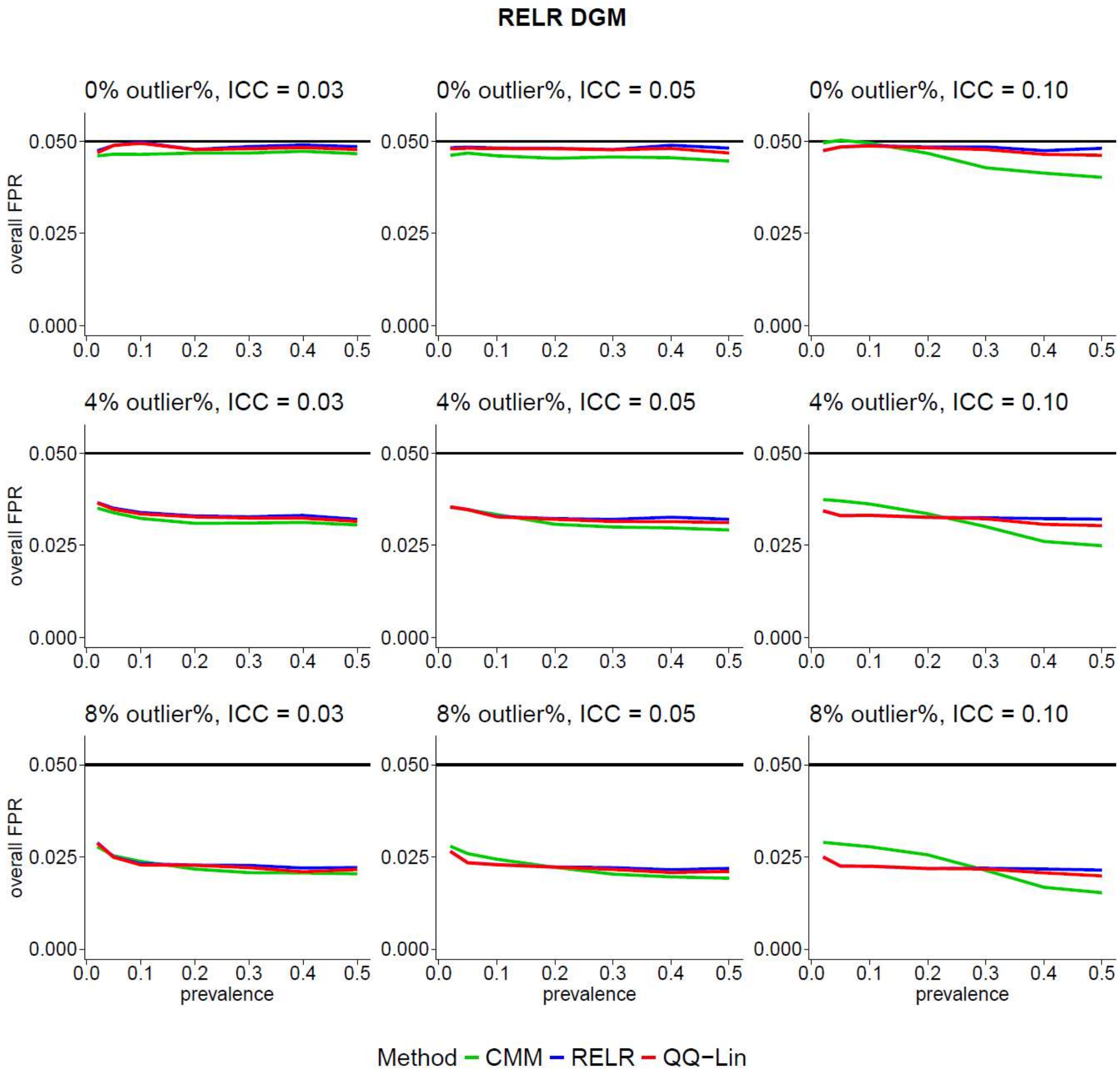

Figure S3 Separate false positive rate (FPR) for good and bad outliers under CMM DGM when there are 100 units of size 1000, across scenarios varying in outlier percentage and overdispersion factor φ. The horizontal black lines represent the nominal level of 0.025 for the separate FPRs.

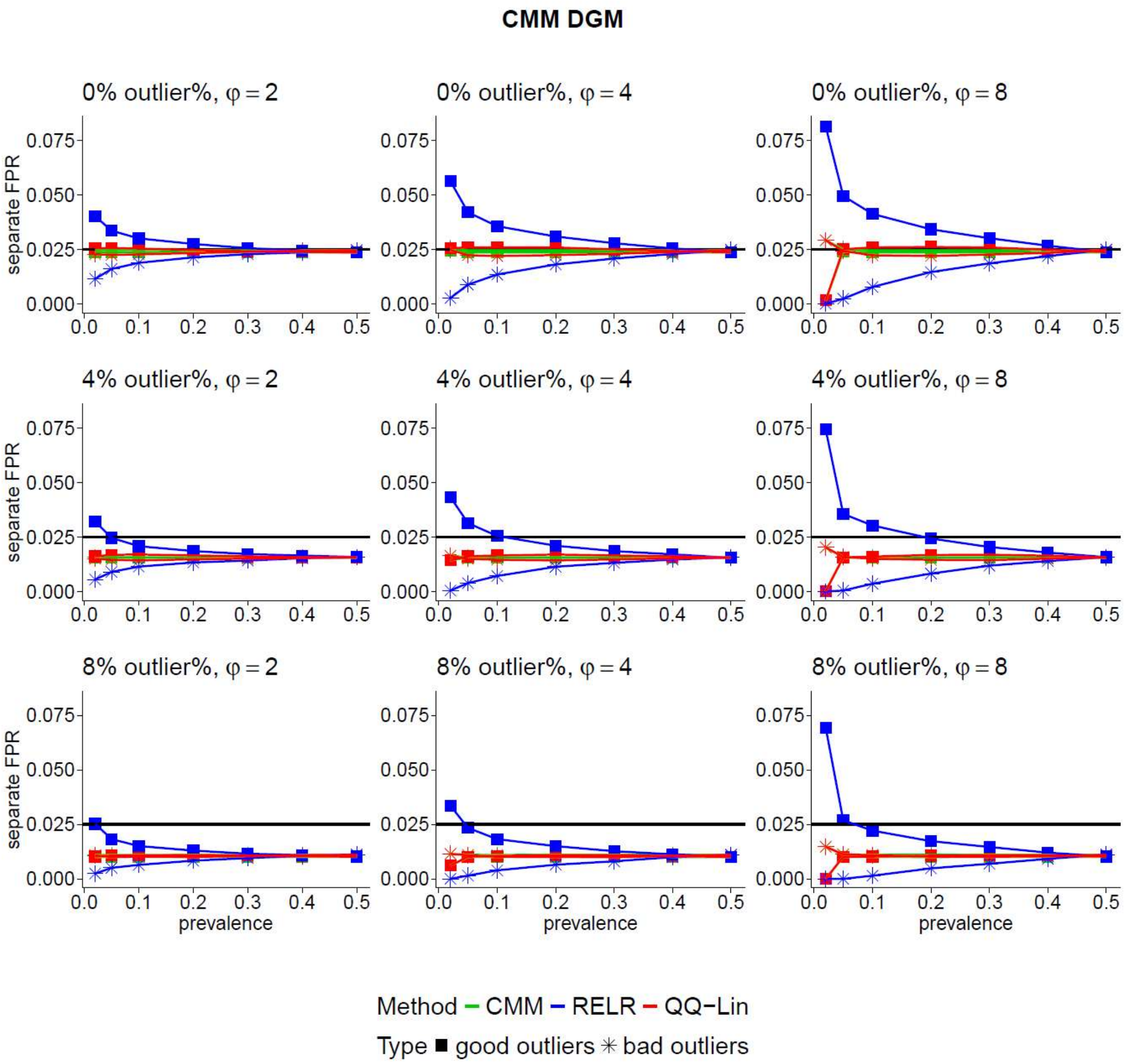

Figure S4 Separate false positive rate (FPR) for good and bad outliers under RELR DGM when there are 100 units of size 1000, across scenarios varying in outlier percentage and intraclass correlation coefficient (ICC). The horizontal black lines represent the nominal level of 0.025 for the separate FPRs.

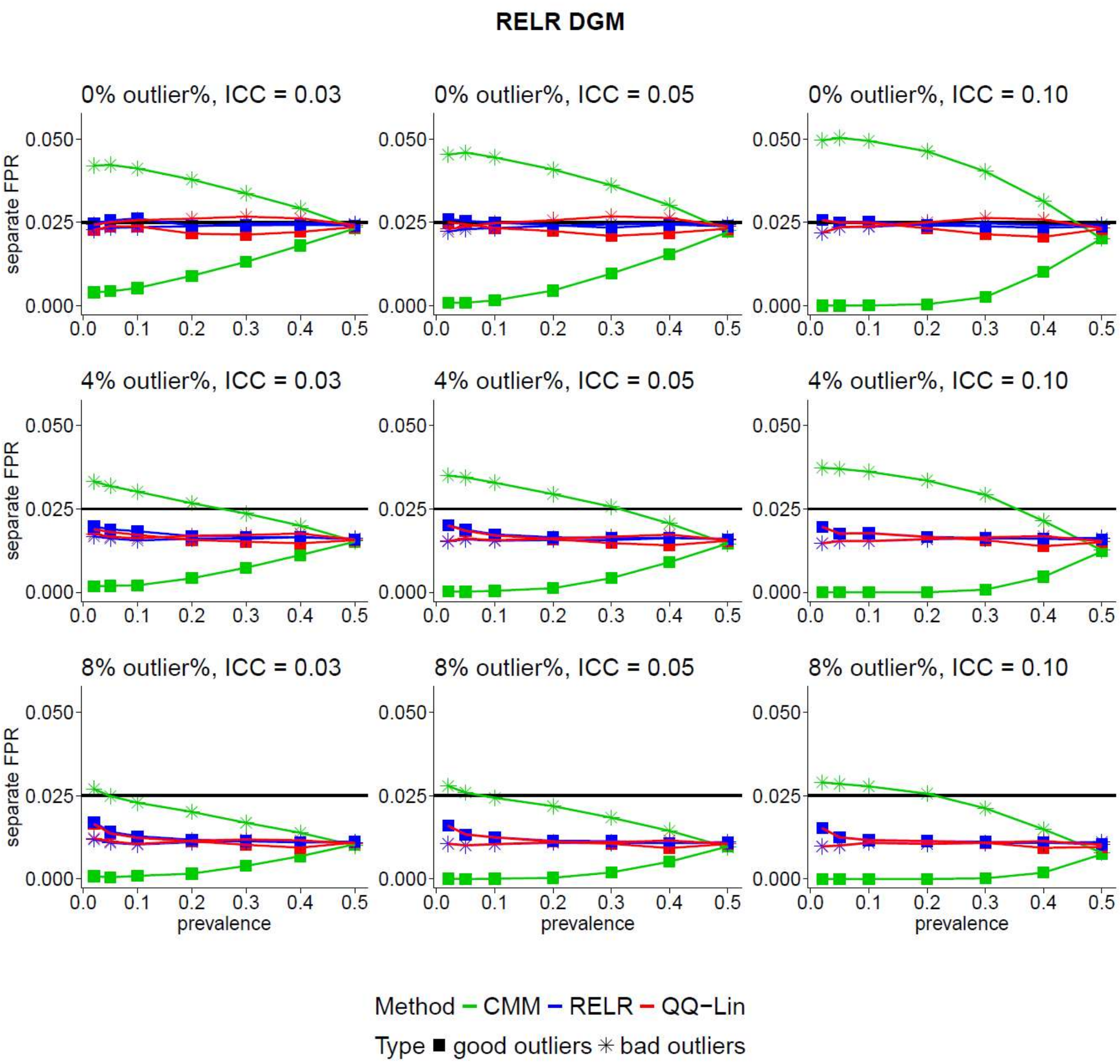

Figure S5 Sensitivity for detection of outliers under CMM DGM when there are 100 units of size 1000, across scenarios varying in outlier percentage and overdispersion factor φ.

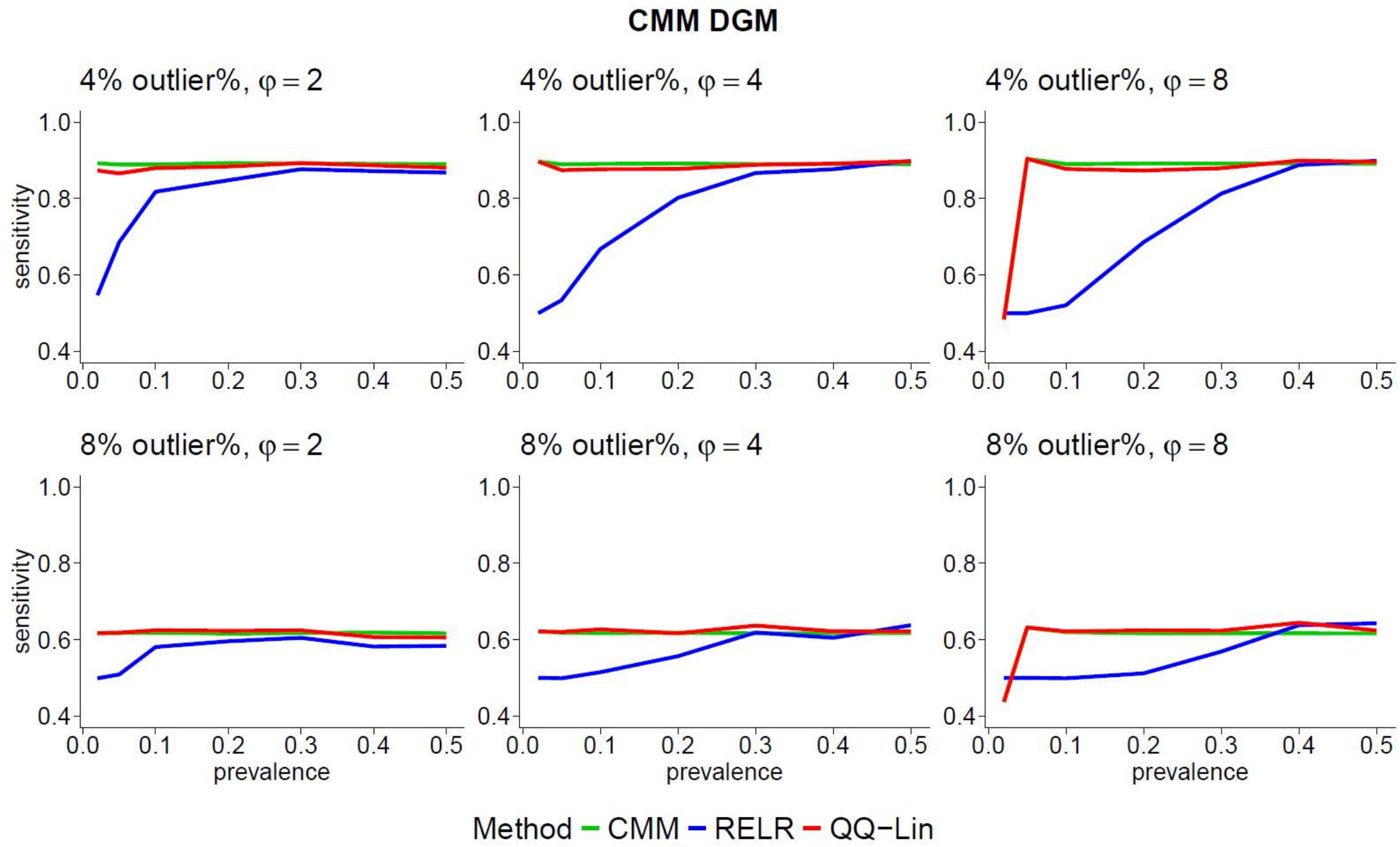

Figure S6 Sensitivity for detection of outliers under RELR DGM when there are 100 units of size 1000, across scenarios varying in outlier percentage and intraclass correlation coefficient (ICC).

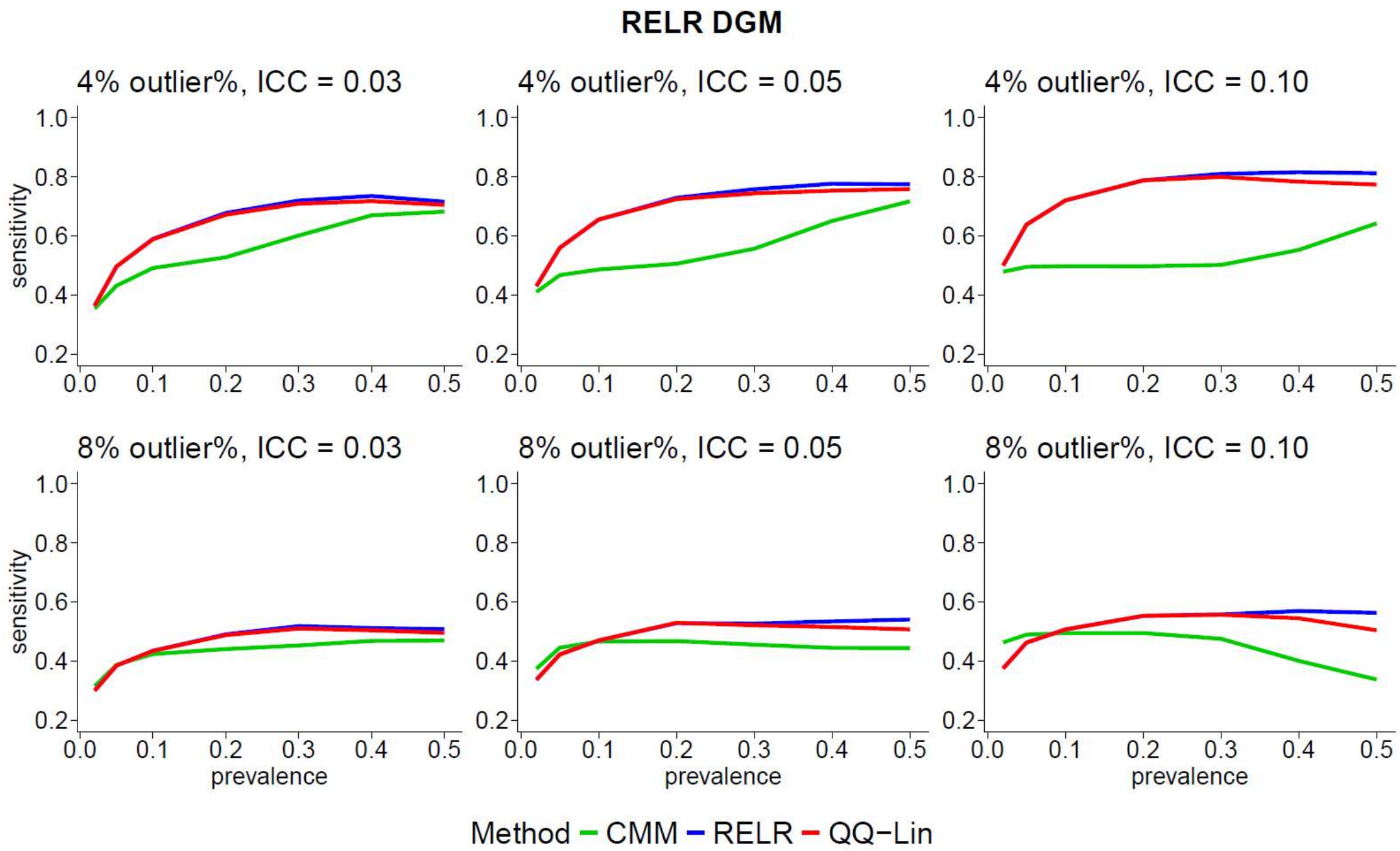

Proportion of correct selections by QQ-Lin (Table S1 and S2)

Table S1 Proportion of correctly selected methods by QQ-Lin when the DGM is the CMM, across scenarios varying in numbers of units, unit sizes, outlier proportions, outcome prevalence, and overdispersion factor.

(A) Overdispersion = 4										(B) Overdispersion = 8									
Outlier%	No. of units	Unit size	Prevalence 0.02	0.05	0.10	0.20	0.30	0.40	0.50	Outlier%	No. of units	Unit size	Prevalence 0.02	0.05	0.10	0.20	0.30	0.40	0.50
0%	50	500	0.92	0.87	0.81	0.69	0.62	0.56	0.71	0%	50	500	0.96	0.96	0.88	0.78	0.67	0.59	0.66
		1000	0.90	0.83	0.73	0.63	0.58	0.56	0.65			1000	0.97	0.89	0.83	0.70	0.62	0.56	0.64
		2000	0.85	0.75	0.65	0.60	0.55	0.54	0.62			2000	0.93	0.84	0.75	0.64	0.59	0.53	0.67
	100	500	0.98	0.95	0.86	0.74	0.63	0.58	0.78		100	500	0.99	0.99	0.96	0.84	0.72	0.61	0.68
		1000	0.97	0.86	0.78	0.66	0.59	0.55	0.73			1000	1.00	0.97	0.87	0.75	0.64	0.57	0.66
		2000	0.91	0.80	0.69	0.61	0.57	0.54	0.69			2000	0.99	0.90	0.80	0.67	0.60	0.55	0.66
	200	500	1.00	0.99	0.95	0.81	0.70	0.62	0.80		200	500	1.00	1.00	1.00	0.95	0.79	0.67	0.67
		1000	1.00	0.96	0.87	0.72	0.64	0.60	0.78			1000	1.00	1.00	0.97	0.84	0.71	0.62	0.67
		2000	0.99	0.89	0.76	0.66	0.60	0.56	0.76			2000	1.00	0.99	0.91	0.74	0.64	0.59	0.63
4%	50	500	0.95	0.97	0.89	0.78	0.67	0.60	0.74	4%	50	500	0.95	1.00	0.99	0.87	0.76	0.64	0.69
		1000	1.00	0.91	0.82	0.70	0.61	0.58	0.63			1000	0.99	1.00	0.91	0.79	0.68	0.59	0.66
		2000	0.94	0.82	0.73	0.64	0.60	0.55	0.62			2000	1.00	0.92	0.83	0.72	0.62	0.57	0.68
	100	500	0.99	1.00	0.95	0.82	0.70	0.63	0.82		100	500	0.99	1.00	1.00	0.93	0.81	0.68	0.72
		1000	1.00	0.95	0.88	0.72	0.65	0.59	0.69			1000	1.00	1.00	0.96	0.86	0.71	0.62	0.70
		2000	0.99	0.90	0.76	0.65	0.61	0.56	0.68			2000	1.00	0.97	0.91	0.75	0.64	0.57	0.64
	200	500	1.00	1.00	0.99	0.91	0.78	0.68	0.82		200	500	1.00	1.00	1.00	0.99	0.90	0.74	0.72
		1000	1.00	1.00	0.96	0.82	0.71	0.64	0.75			1000	1.00	1.00	1.00	0.93	0.80	0.68	0.71
		2000	1.00	0.97	0.87	0.74	0.67	0.62	0.77			2000	1.00	1.00	0.97	0.83	0.72	0.62	0.52
		500	0.97	1.00	0.95	0.79	0.70	0.61	0.70			500	0.95	1.00	1.00	0.93	0.79	0.67	0.68

8%	50	1000	1.00	0.96	0.86	0.72	0.64	0.59	0.66	8%	50	1000	1.00	1.00	0.96	0.82	0.71	0.61	0.66
		2000	0.99	0.88	0.77	0.65	0.59	0.55	0.63			2000	1.00	0.97	0.88	0.74	0.65	0.55	0.54
	100	500	1.00	1.00	0.98	0.87	0.75	0.65	0.71		100	500	0.99	1.00	1.00	0.97	0.87	0.72	0.71
		1000	1.00	0.99	0.92	0.79	0.68	0.61	0.71			1000	1.00	1.00	0.99	0.89	0.77	0.64	0.67
		2000	1.00	0.93	0.84	0.70	0.62	0.59	0.69			2000	1.00	0.99	0.92	0.81	0.69	0.58	0.40
	200	500	1.00	1.00	1.00	0.95	0.84	0.69	0.65		200	500	1.00	1.00	1.00	1.00	0.94	0.78	0.70
		1000	1.00	1.00	0.98	0.87	0.78	0.66	0.76			1000	1.00	1.00	1.00	0.96	0.86	0.71	0.69
		2000	1.00	0.99	0.93	0.80	0.69	0.65	0.78			2000	1.00	1.00	0.98	0.90	0.78	0.63	0.19

Table S2 Proportion of correctly selected methods by QQ-Lin when the DGM is RELR, across scenarios varying in numbers of units, unit sizes, outlier proportions, outcome prevalence, and Intraclass Correlation Coefficient (ICC).

**(A) ICC = 0.03**										**(B) ICC = 0.10**									
Outlier%	No. of units	Unit size	Prevalence 0.02	0.05	0.10	0.20	0.30	0.40	0.50	Outlier%	No. of units	Unit size	Prevalence 0.02	0.05	0.10	0.20	0.30	0.40	0.50
0%	50	500	0.87	0.84	0.84	0.78	0.72	0.60	0.43	0%	50	500	0.97	0.98	0.96	0.90	0.84	0.71	0.55
		1000	0.86	0.87	0.82	0.76	0.70	0.61	0.43			1000	0.97	0.97	0.96	0.91	0.84	0.71	0.54
		2000	0.87	0.85	0.83	0.79	0.71	0.62	0.44			2000	0.98	0.97	0.95	0.92	0.84	0.72	0.55
	100	500	0.94	0.93	0.91	0.85	0.78	0.65	0.47		100	500	1.00	1.00	1.00	0.98	0.92	0.79	0.63
		1000	0.93	0.93	0.90	0.86	0.76	0.64	0.46			1000	1.00	1.00	0.99	0.97	0.91	0.79	0.62
		2000	0.94	0.93	0.91	0.85	0.75	0.65	0.44			2000	1.00	1.00	1.00	0.98	0.92	0.79	0.59
	200	500	0.99	0.99	0.98	0.94	0.82	0.68	0.55		200	500	1.00	1.00	1.00	1.00	0.98	0.91	0.76
		1000	1.00	0.99	0.98	0.93	0.85	0.70	0.52			1000	1.00	1.00	1.00	1.00	0.99	0.90	0.74
		2000	0.99	0.99	0.98	0.95	0.84	0.70	0.53			2000	1.00	1.00	1.00	1.00	0.98	0.89	0.74
4%	50	500	0.88	0.87	0.87	0.83	0.77	0.62	0.40	4%	50	500	1.00	1.00	1.00	0.97	0.90	0.72	0.52
		1000	0.92	0.91	0.89	0.84	0.74	0.61	0.39			1000	1.00	1.00	1.00	0.98	0.89	0.75	0.54
		2000	0.94	0.92	0.93	0.85	0.76	0.62	0.41			2000	1.00	1.00	1.00	0.98	0.92	0.75	0.55
	100	500	0.96	0.97	0.96	0.92	0.84	0.68	0.47		100	500	1.00	1.00	1.00	0.99	0.96	0.83	0.65
		1000	0.96	0.97	0.95	0.92	0.84	0.69	0.47			1000	1.00	1.00	1.00	1.00	0.96	0.84	0.64
		2000	0.97	0.97	0.96	0.91	0.82	0.66	0.45			2000	1.00	1.00	1.00	1.00	0.96	0.83	0.63
	200	500	1.00	0.99	0.99	0.97	0.91	0.74	0.57		200	500	1.00	1.00	1.00	1.00	1.00	0.93	0.82
		1000	1.00	1.00	1.00	0.98	0.92	0.76	0.57			1000	1.00	1.00	1.00	1.00	1.00	0.93	0.79
		2000	1.00	1.00	0.99	0.98	0.91	0.75	0.56			2000	1.00	1.00	1.00	1.00	1.00	0.93	0.81
		500	0.92	0.92	0.93	0.85	0.79	0.66	0.46			500	1.00	1.00	1.00	0.99	0.92	0.78	0.59

8%	50	1000	0.93	0.94	0.92	0.87	0.78	0.63	0.47	8%	50	1000	1.00	1.00	1.00	0.99	0.93	0.77	0.58
		2000	0.95	0.94	0.95	0.90	0.79	0.66	0.46			2000	1.00	1.00	1.00	0.99	0.93	0.76	0.58
	100	500	0.97	0.98	0.96	0.94	0.89	0.74	0.58		100	500	1.00	1.00	1.00	1.00	0.98	0.88	0.76
		1000	0.98	0.98	0.98	0.96	0.88	0.71	0.55			1000	1.00	1.00	1.00	1.00	0.99	0.87	0.71
		2000	0.99	0.99	0.99	0.96	0.88	0.70	0.53			2000	1.00	1.00	1.00	1.00	0.98	0.87	0.70
	200	500	0.99	1.00	0.99	0.99	0.94	0.82	0.73		200	500	1.00	1.00	1.00	1.00	1.00	0.97	0.90
		1000	1.00	1.00	1.00	0.99	0.95	0.82	0.73			1000	1.00	1.00	1.00	1.00	1.00	0.95	0.89
		2000	1.00	1.00	1.00	0.99	0.95	0.80	0.71			2000	1.00	1.00	1.00	1.00	1.00	0.97	0.85

Overall agreement rates between the CMM and RELR approaches (Table S3)

Table S3 Mean (standard deviation) agreement rates among all units between the CMM and RELR approaches (500 simulations; 100 units of size 1000).

CMM DGM								
Overdispersion factor	Outlier%	Prevalence						
		0.02	0.05	0.10	0.20	0.30	0.40	0.50
2	0%	0.97 (0.02)	0.98 (0.01)	0.99 (0.01)	0.99 (0.01)	1.00 (0.01)	1.00 (0.00)	1.00 (0.00)
	4%	0.96 (0.02)	0.97 (0.02)	0.98 (0.02)	0.99 (0.01)	0.99 (0.01)	1.00 (0.01)	1.00 (0.01)
	8%	0.94 (0.03)	0.95 (0.03)	0.96 (0.03)	0.98 (0.02)	0.99 (0.02)	0.99 (0.01)	1.00 (0.01)
4	0%	0.95 (0.02)	0.97 (0.02)	0.98 (0.01)	0.99 (0.01)	0.99 (0.01)	1.00 (0.01)	1.00 (0.00)
	4%	0.94 (0.02)	0.95 (0.02)	0.97 (0.02)	0.98 (0.01)	0.99 (0.01)	1.00 (0.01)	1.00 (0.00)
	8%	0.92 (0.03)	0.94 (0.03)	0.95 (0.03)	0.96 (0.03)	0.98 (0.02)	0.99 (0.02)	1.00 (0.01)
8	0%	0.89 (0.02)	0.95 (0.02)	0.97 (0.02)	0.98 (0.01)	0.99 (0.01)	0.99 (0.01)	1.00 (0.00)
	4%	0.87 (0.02)	0.95 (0.02)	0.96 (0.02)	0.97 (0.02)	0.98 (0.01)	0.99 (0.01)	1.00 (0.00)
	8%	0.85 (0.02)	0.93 (0.03)	0.94 (0.03)	0.95 (0.03)	0.97 (0.03)	0.98 (0.02)	1.00 (0.01)

RELR DGM								
Intraclass correlation coefficient	Outlier%	Prevalence						
		0.02	0.05	0.10	0.20	0.30	0.40	0.50
0.03	0%	0.96 (0.02)	0.96 (0.02)	0.96 (0.02)	0.97 (0.02)	0.98 (0.01)	0.99 (0.01)	1.00 (0.00)

	4%	0.96	0.96	0.96	0.96	0.97	0.99	1.00
		(0.02)	(0.02)	(0.02)	(0.02)	(0.02)	(0.01)	(0.01)
	8%	0.95	0.95	0.95	0.95	0.96	0.98	1.00
		(0.02)	(0.02)	(0.02)	(0.02)	(0.02)	(0.01)	(0.01)
0.05	0%	0.95	0.95	0.96	0.96	0.97	0.99	1.00
		(0.02)	(0.02)	(0.02)	(0.02)	(0.02)	(0.01)	(0.01)
	4%	0.95	0.95	0.95	0.96	0.96	0.98	0.99
		(0.02)	(0.02)	(0.02)	(0.02)	(0.02)	(0.01)	(0.01)
	8%	0.94	0.94	0.94	0.94	0.95	0.97	0.99
		(0.02)	(0.02)	(0.02)	(0.02)	(0.02)	(0.02)	(0.01)
0.10	0%	0.95	0.95	0.95	0.95	0.96	0.98	0.99
		(0.02)	(0.02)	(0.02)	(0.02)	(0.02)	(0.01)	(0.01)
	4%	0.95	0.95	0.95	0.95	0.95	0.97	0.99
		(0.02)	(0.02)	(0.02)	(0.02)	(0.02)	(0.02)	(0.01)
	8%	0.94	0.94	0.94	0.94	0.94	0.96	0.98
		(0.03)	(0.02)	(0.02)	(0.02)	(0.02)	(0.02)	(0.01)
